# Treatment of Club-Foot

**Published:** 1891-01-10

**Authors:** 


### TREATMENT OF CLUB-FOOT.

The progress of orthopædic surgery of late years is remarkably illustrated by a record such as that contributed to the *Ceutralbl-f.-Chir.*, of the successive operations or modes of treatment adopted between 1880 and 1889 in the clinic of the late Prof. Volkmann, by his pupil and now successor, Von Büngner. During that period of ten years, 156 cases of congenital club-foot had been admitted, 58 were throughout treated orthopædically, and in 98, some surgical operation was found necessary to complete, or to accelerate the cure.

The operations and the periods during which they were practised, were :

| Operation. | No. of times performed. | No. of individuals operated upon. | Of ages between | Period in which performed. |
|---|---|---|---|---|
| | | | Y. Mo. Y. Mo. | |
| Tenotomy of Tendo Achillis ... | 32 | 24 | — 6 to 29 — | 1880-1889 |
| Tenotomy of Tendon of Tib. post . | 8 | 7 | — 6 to 14 — | 1880-1882 |
| Tenotomy of both Tendons ... ... | 8 | 6 | 2, 6 to 15 — | 1880-1882 |
| Osteotomies of Tarsus ... ... ... | 4 | 3 | 8 — to 15 — | 1880-1882 |
| Excision of Talus .. | 25 | 17 | 1, 3 to 26 — | 1884-1888 |
| Phelps' Operation... | 21 | 14 | — 4 to 15 — | 1884-1889 |

Tenotomies, except of the tendo Achillis, were abandoned fter the year 1882, their results having been rarely found satisfactory or permanent. Excision of the talus was discontinued in 1888, after four years' experience, the normal relation of the parts having been restored in one-third only of the cases, and then only after long orthopædic treatment. In another third the improvement was scarcely perceptible, and in the remainder no benefit whatever followed. Phelps' operation, on the other hand, which was begun about the same time, has more than fulfilled the high expectations formed of it, having been in almost every instance perfectly successful, and now takes its place as the chief operation. If cases of equinovarus are brought to the hospital within the first three months, the mother is instructed to practise, with steadily increasing force, the manipulation of the foot into the opposite or prone position on every occasion of dressing or otherwise attending to the infant. When these movements have been practised perseveringly till the child is a year or eighteen months old and begins to walk, a Scarpa's shoe is given, and generally succeeds in enabling the child to stand and walk with the sole applied to the ground. Massage of the muscles with stimulating liniments is often of service, but bandages are not considered appropriate at so early an age. If not seen until the second year, similar but much more vigorous manipulations in the way of abduction and pronation are employed, and, these failing, Phelps' operation is performed. In slighter cases, repeated fixation of the foot in progressively increasing rectification of the position is the usual treatment. The child having been put under chloroform, the foot is held by an assistant in the utmost possible pronation and abduction, the tendo Achillis being divided if it present much resistance. A bandage of plaster of Paris alone, or combined with water-glass, is then carried from the base of the toes to the knee, or, if thought advisable, nearly to the hip, and left on for two or three weeks. It is then removed, and a further rectification of the position of the foot having been effected as before, the bandages are again applied. This procedure is gone through at regular intervals until every trace of deformity has disappeared, when the child is allowed to walk with a Scarpa's shoe, which is worn for a year, or even longer, should there appear to be any tendency to a relapse. But by far the greatest advance made at Halle is the treatment of cases of club-foot by the so-called portable water-glass bandage, the use of which is based on the theory maintained by Volkmann that apparent relapses are due to the statical equilibrium between the load and the support not having been perfectly restored. So long as this abnormal relation subsists the use of the limb must tend to further displacement, but when once brought into absolute correspondence the tendency ceases, since the effect cannot exist without the cause. He also held that, however advanced the age of the patient, and complete the ossification of the bones, there existed still almost unimpaired a "transformative power," or a power of moulding the opposed surfaces of the bones by growth and absorption, in the ordinary process of nutrition, to the conditions under which they were placed for the time being. He and his pupils have thus achieved complete success in the treatment, without any surgical operation, of the very worst cases of congenital club-foot in adults from 20 to 50 years of age, cases which all previous experience would have pronounced utterly hopeless. One patient, æt. 24, was a case of extreme congenital double club-foot, for which no treatment had ever been attempted. The soles were turned upward and backward, the dorsum of the foot resting on the ground, above which the toes, pointing inwards and backwards, were raised a full hand's breadth. The treatment—consisting of forcible flexion under chloroform, and the application of a water-glass bandage, with which the patient was permitted to walk, thus continuing the pressure in the desired direction—was begun in May, 1887, and carried on with the utmost care and perseverance. In July he could walk without a stick or any support other than the boot-like bandage, and returned home. In November the application was repeated, and by the end of the following July, or fourteen months from the commencement of the treatment, the cure was complete. The man wore ordinary boots, could walk for an hour without fatigue, and when wearing socks, the feet did not show the smallest indication of the previous deformity. Others, aged respectively, 24, 32, and 49 years, have given results no less astonishing; in fact von Büngner believes that there is no case, however bad or advanced in life, that will not yield to a force which, in combining mechanical pressure with the formative efforts of nature, is absolutely irresistible. It is not suited for the tender limbs of infants, though the same in principle as the treatment he employs in the case of those of over a year; but, while it is the only possible resource in those of adults, the conditions of their life conduce to, and co-operate actively in bringing about, the desired changes.

